# Enhancement of Executive Functions but Not Memory by Multidomain Group Cognitive Training in Patients with Parkinson's Disease and Mild Cognitive Impairment: A Multicenter Randomized Controlled Trial

**DOI:** 10.1155/2020/4068706

**Published:** 2020-11-30

**Authors:** Elke Kalbe, Ann-Kristin Folkerts, Anja Ophey, Carsten Eggers, Saskia Elben, Karina Dimenshteyn, Patricia Sulzer, Claudia Schulte, Nele Schmidt, Christian Schlenstedt, Daniela Berg, Karsten Witt, Lars Wojtecki, Inga Liepelt-Scarfone

**Affiliations:** ^1^Medical Psychology∣Neuropsychology and Gender Studies & Center for Neuropsychological Diagnostics and Interventions (CeNDI), Faculty of Medicine and University Hospital Cologne, University of Cologne, Cologne, Germany; ^2^Department of Neurology, University Hospital of Marburg, Center for Mind,Brain and Behavior—CMBB, Universitäten Marburg und Gießen, Marburg, Germany; ^3^Department of Neurology, Faculty of Medicine and University Hospital Cologne, University of Cologne, Cologne, Germany; ^4^Department of Neurology, Center for Movement Disorders and Neuromodulation and Institute of Clinical Neuroscience and Medical Psychology, Medical Faculty, Heinrich-Heine-University Duesseldorf, Duesseldorf, Germany; ^5^German Center for Neurodegenerative Diseases (DZNE) and Hertie Institute for Clinical Brain Research, Department of Neurodegenerative Diseases, University of Tuebingen, Tuebingen, Germany; ^6^Department of Neurology, University Hospital Schleswig-Holstein, Christian-Albrechts-University, Kiel, Germany; ^7^Research Center Neurosensory Science, Carl von Ossietzky University Oldenburg, Oldenburg, Germany; ^8^Department of Neurology, Hospital zum Heiligen Geist, Kempen, Frankfurt, Germany

## Abstract

**Background:**

Meta-analyses have demonstrated cognitive training (CT) benefits in Parkinson's disease (PD) patients. However, the patients' cognitive status has only rarely been based on established criteria. Also, prediction analyses of CT success have only sparsely been conducted.

**Objective:**

To determine CT effects in PD patients with mild cognitive impairment (PD-MCI) on cognitive and noncognitive outcomes compared to an active control group (CG) and to analyze CT success predictors.

**Methods:**

Sixty-four PD-MCI patients (age: 67.61 ± 7.70; UPDRS-III: 26.58 ± 13.54; MoCA: 24.47 ± 2.78) were randomized to either a CT group or a low-intensity physical activity CG for six weeks (twice weekly, 90 minutes). Outcomes were assessed before and after training. MANOVAs with follow-up ANOVAs and multiple regression analyses were computed.

**Results:**

Both interventions were highly feasible (participation, motivation, and evaluation); the overall dropout rate was 4.7%. Time × group interaction effects favoring CT were observed for phonemic fluency as a specific executive test (*p*=0.018, *η*_*p*_^2^=0.092) and a statistical trend for overall executive functions (*p*=0.095, *η*_*p*_^2^=0.132). A statistical trend for a time × group interaction effect favoring CG was shown for the digit span backward as a working memory test (*p*=0.098, *η*_*p*_^2^=0.043). Regression analyses revealed cognitive baseline levels, education, levodopa equivalent daily dose, motor scores, and ApoE status as significant predictors for CT success.

**Conclusions:**

CT is a safe and feasible therapy option in PD-MCI, yielding executive functions improvement. Data indicate that vulnerable individuals may show the largest cognitive gains. Longitudinal studies are required to determine whether CT may also attenuate cognitive decline in the long term. This trial is registered with DRKS00010186.

## 1. Introduction

In Parkinson's disease (PD), cognitive impairment is a common nonmotor symptom. The prevalence of mild cognitive impairment in PD (PD-MCI) is approximately 40%; about 32% of patients show cognitive impairment at the time of diagnosis [1]. Dysfunctions in executive function and memory are the most frequent symptoms in PD-MCI, but patients may also suffer from deficits in attention, visuocognition, and also language [2, 3]. Cognitive impairment has an enormous impact on PD patients' and their relatives' quality of life (QoL) [4, 5], increases patients' mortality [6], and has significant health-economic consequences [7]. Because no drug-based prevention and limited pharmacological options for the treatment of cognitive impairment in PD exist [8], nonpharmacological interventions (e.g., cognitive training (CT) and physical interventions) are increasingly recognized.

Two meta-analyses have demonstrated CT benefits in PD patients, particularly with respect to executive functions, working memory, memory, and processing speed [9, 10]. However, a recent Cochrane review and meta-analysis [11] found that studies on CT in PD patients so far do not give evidence for “important cognitive improvements.” The authors point out that reliable conclusions cannot be drawn yet due to a small number of studies with few participants, limitations of study design and execution, and imprecise results. They emphasize the need for robust studies with patients whose cognitive state is diagnosed with formal criteria. In fact, in CT studies, the patients' cognitive status has only rarely been assessed based on established criteria. Therefore, its efficacy in PD, PD-MCI, and PD dementia (PDD) cannot be derived [11, 12]. Also, noncognitive outcomes (e.g., quality of life and neuropsychiatric symptoms) have only scarcely been examined in existing randomized controlled trials (RCTs) [9, 11]. Furthermore, to target CT to patients' specific profiles, predictors for CT responsiveness should be defined. Studies including healthy elderly people and (non-PD) MCI patients have demonstrated that age, education, ApoE, and cognitive status at baseline [13–18] are predictors of positive CT responsiveness. For PD, results are ambiguous, reporting that lower baseline scores and PD-MCI [19, 20], but a higher general cognitive status [21], and longer [22] or shorter [21] PD duration may predict positive training responsiveness.

Thus, the aims of this RCT were (1) to examine the efficacy of a multidomain group CT in contrast to an active control group (CG) in PD-MCI patients and (2) to analyze sociodemographic, clinical, neuropsychological, and genetic predictors for CT success. We hypothesize that CT is superior to an active CG. Due to the heterogeneous results in the previous literature on predictors of CT responsiveness, regarding our second aim, we state no specific hypotheses and follow an exploratory analysis.

## 2. Materials and Methods

### 2.1. Study Design

The multicenter RCT is registered in the German Clinical Trials Register (DRKS; ID: DRKS00010186; the study registration is outlined as retrospective due to an administrative delay. The first patient was enrolled three months after the registration process was started. A formal confirmation of this process from the German Clinical Trials Register can be obtained from the authors). It was first approved by the ethics committee of the Faculty of Medicine, University of Tuebingen; the other study sites then obtained a second vote by their local ethics committees. The research was conducted in accordance with the Declaration of Helsinki. Prior to data assessment, all participants gave written informed consent. No published study protocol exists. The reporting of this trial follows the CONSORT guidelines [23].

Data collection was conducted in four German University Hospitals (Cologne, Duesseldorf, Tuebingen, Kiel) between July 2016 and May 2018. Here, we only report pre- and posttest data relevant for the aims of this manuscript. 6- and 12-months follow-up data, as well as electroencephalography, blood sampling, and home-based physical behavior assessments with accelerometers will be reported elsewhere. After obtaining written informed consent, participants were screened for eligibility. Afterward, baseline assessment was conducted, and patients were randomized to either CT or CG. Pre- and posttests were scheduled within a 4-week's time frame pre- and postintervention. All assessments and intervention sessions were conducted with patients in the medication ON state; short breaks were offered to avoid excessive strain.

The randomization was conducted by an independent member of the Cologne research group. Randomization lists for allocating patients to CT or CG were prepared with an online tool (http://www.randomizer.org) for each study site which received two lists for two recruitment phases each. These lists contained the allocation of a maximum of ten patients to CT or CG. After a participant was included in the study, a further independent colleague at each study site conducted the allocation with the help of the list (i.e., the first patient was allocated to the first list position).

The intervention facilitators were not blinded to group allocation. The patients and relatives who were involved in external ratings were blinded because they were not aware about the targeted study intervention. The outcome assessors were blinded for the intervention type; patients were asked to give no information about their group allocation during the assessments.

Data management was supported by an online database which was only accessible through a specially secured Internet connection provided by the German Center for Neurodegenerative Diseases (DZNE), Bonn. It was thus possible to enter pseudonymized data from all study sites. This was performed by two members according to the dual control principle. The data monitoring was defined within a detailed manual and was conducted by two members of another study site.

### 2.2. Patient Recruitment and Eligibility Criteria

Some participants were recruited personally after checking for eligibility criteria in patients' files. Others who had given their consent to be informed about new studies were contacted by telephone. Furthermore, the study was presented in PD social support groups and on a Cologne patients' symposium.

Inclusion criteria were (i) age between 50 and 80 years, (ii) PD diagnosis according to the UK Brain Bank Criteria [24], (iii) self-reported cognitive impairment assessed with the subjective cognitive impairment (SCI) questionnaire [25] and/or objective cognitive impairment assessed with the Montreal Cognitive Assessment (MoCA) [26] <26 points, (iv) PD-MCI according to Movement Disorders Society (MDS) Task Force Level-II criteria [25] (cognitive impairment in at least two cognitive tests; *z*-score ≤ −1 SD below the mean normative score), (v) PD duration ≥three years, (vi) stable medication within four weeks before screening, and (vii) written informed consent.

Exclusion criteria were (i) clinical PDD diagnosis or outcomes in the Pill-Questionnaire [27] indicating impaired activities of daily living (ADL), (ii) depression (Beck Depression Inventory II (BDI-II; [28]) ≥20 points), (iii) acute suicide tendency, (iv) severe comorbidities affecting life expectancy, medication, or QoL, (v) severe fatigue (self-disclosure in the anamnesis and doctor's letters), (vi) prominent impulse control disorder or dopamine dysregulation syndrome (self-disclosure in the anamnesis and doctor's letters), (vii) acute psychosis or psychotic episode in the last six months, (viii) dementia medication, (ix) participation in other treatment studies within the last two months, (x) deep brain stimulation (DBS) to exclude any DBS effects, and (xi) pregnancy or the nursing period.

### 2.3. Interventions

Both interventions included two sessions per week for 90 minutes over six weeks and were conducted in groups of three to five individuals (Supplementary Table 1 for details). For CT, the standardized NEUROvitalis program [29] targeting executive functions, memory, attention, and visuocognition was used. Each session is characterized by several training elements: psychoeducation (e.g., possible cognitive decline in PD, memory strategies, and risk and protective factors for cognitive aging), group tasks and activity games, individual exercises, and homework. For CG, a low-intensity physical activity program was developed by a sports scientist (author CS) which aimed to be beneficial for PD patients but to have minimal effects on cognition. Main trained domains are stretching, flexibility, loosening up, and relaxation. Each session starts with a warm up exercise, followed by specific exercises on the four trained domains as well as psychoeducation on PD symptoms and therapy options and homework.

### 2.4. Outcomes

Next to demographic and PD-relevant data, nonpharmacological treatment (physiotherapy and CT) and the self-reported everyday activity level were assessed. At baseline, the General Self-Efficacy Scale (GSE) was also applied (Tables 1 and 2 for all instruments).

Primary study outcomes were memory and executive functions. Secondary outcomes were attention, working memory, visuocognition, language, ADL, self-reported physical activity, depression, QoL, self-experienced attention deficits, and motor impairment including the motor score of the Unified Parkinson's Disease Rating Scale (UPDRS-III) and freezing of gait (FOG) assessed with the Freezing of Gait Questionnaire. Parallel test versions were used if available.

Neuropsychological outcome assessors were trained psychologists; neurological scale assessments were conducted by neurologists, physicians in neurological training, and PD nurses.

At the pretest, blood sampling was obtained for ApoE genotyping: rs429358. DNA was isolated from EDTA blood by the salting out method and stored at 4°C. Genetic testing was performed at the Hertie Institute for Clinical Brain Research Tuebingen. Genotyping was performed using the multiplex SNaPshot analysis (3500xl Applied Biosystems, Foster City, CA, USA; Software: GeneMapper) with a capillary electrophoresis approach. Primers and conditions are available upon request.

Feasibility of trainings were assessed by (i) patient participation, (ii) a training diary, in which patients rated their current training motivation (6-point Likert scale from 0 = “not motivated” to 6 = “very motivated”) and whether and how long they had trained at home, (iii) a question after each session how patients liked today's session (6-point Likert scale from 0 = “not good at all” to 6 = “very good”), (iv) a school grade given by each patient after the last session from 1 = “very good” to 6 = “insufficient” for the overall training, and (v) the question whether they would recommend it.

### 2.5. Statistical Analysis

The power analysis conducted in G*∗*power 3.1 was based on previously published studies [30, 31] and a meta-analysis [9] reporting small overall effect sizes of CT in PD and medium to large effect sizes on executive functions and working memory. On the basis of these results and because this study only included PD-MCI patients, for which larger effect sizes were found in previous literature [19], medium effect sizes were expected across cognitive domains (*d* ≥ 0.5). With a power of 80% and a significance level of *p*=0.05, *n* = 34 patients should be included in the two groups. Considering a dropout rate of 10%–15% [31], the sample calculation provided an overall sample size of *n* = 80 patients.

**Figure 1:**
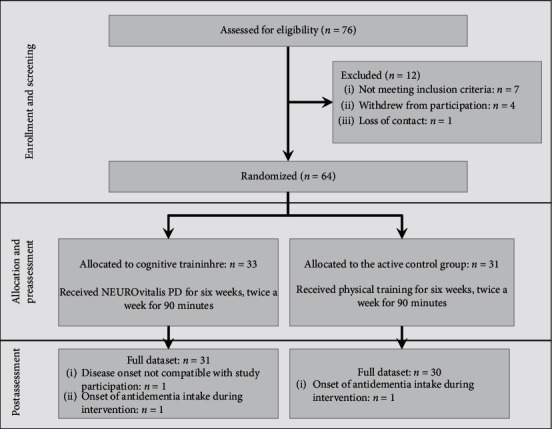
Participation flow.

SPSS 25 statistics software (IBM) was used for data analyses. Analyses were carried out using both an intention-to-treat (ITT) and a per-protocol (PP) approach. For ITT analyses, we also considered data of *n* = 3 patients who dropped out during the intervention period (Figure 1). Missing data were imputed using the last observation carried forward (LOCF) method where possible. For the PP analysis, only patients who completed the pre- and postassessment (*n* = 61) were included in the analyses, and no imputation methods were used. The alpha level was set at 0.05 for all analyses. Furthermore, *p* values up to an alpha level ≤0.10 were regarded as statistical trends worth reporting considering the active CG implementation. Only significant results and statistical trends are reported in the text.

For the baseline comparison between groups, variables were previously tested for normal distribution with the Kolmogorov–Smirnov test. Mean scores and standard deviations, median and range, or frequencies with percentages are indicated, as appropriate. To compare both groups, two-sample *t*-tests or the Mann–Whitney *U* test were conducted, as appropriate. Chi-square statistics were used for comparing frequencies.

To compare training effects between the groups, 2 × 2 (time × group) mixed multivariate analysis of variance (MANOVAs) was used to test for overall domain specific effects for all cognitive domains including global cognition considering all cognitive tests (Table 1). Follow-up analyses of variance (ANOVAs) were conducted for relevant outcomes. Partial eta square (*η*_*p*_^2^) is reported as the effect size, indicating small (0.01 ≤ *η*_*p*_^2^ ≤ 0.06), medium (0.06 ≤ *η*_*p*_^2^ < 0.14), or large (*η*_*p*_^2^ ≥ 0.14) effects [32]. Test scores were previously standardized into *z*-values using published normative data.

To exploratory analyze predictors of intervention responsiveness, multiple regression analyses were performed for the CT group and, to control for specificity, for CG. As dependent variables, change scores (Δ = post-pre) for single outcomes and overall domains (mean change score across assigned tests) were computed. Following previous reports, predictor variables included were age, education, baseline level of the respective cognitive and noncognitive scores, levodopa equivalent daily dose (LEDD) [33], UPDRS-III, and ApoE state. The assumptions of multiple regressions were checked according to Field [34].

## 3. Results

76 patients were screened for eligibility, and after the pretest, 64 patients were randomly allocated to the CT group (*n* = 33) or CG (*n* = 31), respectively. Three participants (CT: *n* = 2, CG: *n* = 1) dropped out (4.7%) during the intervention phase. Therefore, *n* = 61 patients were included in PP and *n* = 64 patients in ITT analyses (Figure 1).

Both groups were comparable with regard to all demographic and clinical variables, overall cognitive state, and ApoE state (Table 3). Hoehn and Yahr PD disease stage was moderate in most patients. Both groups indicated minimal depressive symptoms, with 21.9% of patients taking antidepressants.

### 3.1. Training Results

Regarding feasibility, the groups did not differ in participation, training motivation, overall training grade, and training recommendation (Supplementary Table 2). Patients in both groups attended on average 11 (range 8–12) training sessions, were highly motivated, and evaluated on average the intervention with the grade 2 (“good”). All participants would recommend the training. CG participants were more satisfied after training sessions than CT participants (*x̅* = 5 vs. *x̅* = 4; max. 6 = “very good”). However, the CT group continued to train at home significantly more frequently (*x̅* = 9 vs. *x̅* = 6 of 11 sessions).

One adverse event not related to the intervention occurred in the CT group; the patient fell at home, resulting in a head laceration. One adverse event related to the intervention occurred in CG: one participant fell during the intervention due to balance problems, resulting in a cut over the left eyebrow and hip and chest pain. Both patients continued participation.

**Figure 2:**
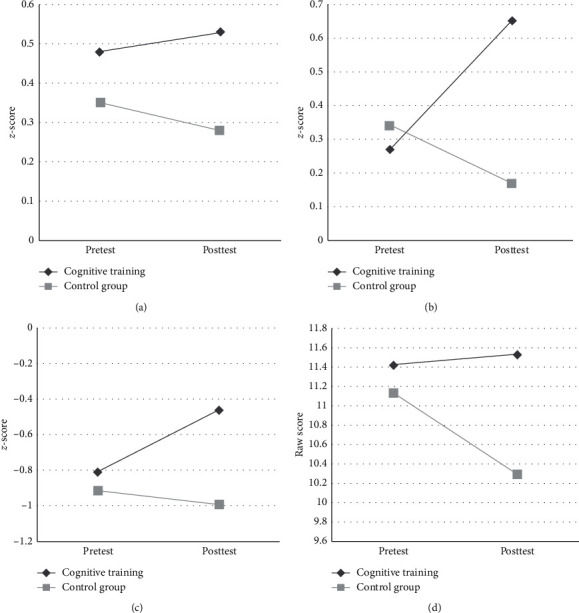
Executive function *z*-scores at the pre- and posttest for both training groups. Error bars are not illustrated for reasons of clarity; the standard deviations are presented in Table 1. MCST = modified Wisconsin card sorting test. (a) Semantic word fluency, (b) phonemic word fluency, (c) MCST categories, and (d) key search.

ITT and PP data are presented in Tables 1 and 2. In the following, only ITT results are presented as the analyses only differ marginally. With regards to effects favoring CT compared to CG, time × group interaction effects were seen in the MANOVA for executive functions (Pillai's trace *V* = 0.132, *F* (4, 55) = 2.088, statistical trend *p*=0.095, *η*_*p*_^2^ = 0.132; Figure 2) and in the ANOVA for phonemic fluency (*F* (1, 58) = 5.901, *p*=0.018, *η*_*p*_^2^ = 0.092), both with medium effect sizes. For self-reported physical activity (Physical Activity Scale for the Elderly, PASE), a small effect size was observed (*F* (1,  62) = 2.741, statistical trend *p*=0.103, *η*_*p*_^2^ = 0.042). A time × group interaction effect with a small effect size favoring CG was seen for the digit span backward task (*F* (1, 62) = 2.816, statistical trend *p*=0.098, *η*_*p*_^2^ = 0.043). No further effects either on neuropsychological or motor symptoms were found.

### 3.2. Prediction Analysis

Table 4 presents results of both the ITT and PP regression analyses for models reaching the statistical significance for both groups. Only ITT results are summarized, as both analyses showed widely consistent results. For the CT group, significant regression models explained between 24.1% and 45.5% of the variance (adjusted *R*^2^).

In the CT group, lower baseline levels significantly predicted positive training responsiveness in executive functions (*β* = −0.446), working memory (*β* = −0.622), visuocognition (*β* = −0.548), and language (*β* = −0.565) as well as in nine single test scores (−0.729 ≤ *β* ≤ −0.398). For higher age, a trend for predicting training positive training responsiveness in the domain of visuocognition (*β* = 0.334) and the phonemic fluency assessment (*β* = 0.405) were found. Lower education showed a trend for predicting positive training responsiveness in motor functioning (UPDRS, *β* = 0.403). Lower UPDRS-III (i.e., better motor functioning) scores at baseline significantly predicted positive training responsiveness in the language domain (*β* = −0.441) and phonemic fluency (*β* = −0.507). A higher LEDD was predictive for positive training responsiveness in semantic (*β* = 0.493) and phonemic (*β* = 0.423) fluency. Finally, carrying the ApoE4 allele might predict a better outcome in the language domain (*β* = 0.463) and phonemic fluency (*β* = 0.473). A reduction of depressive symptoms were predicted by more depressive symptoms at baseline (*β* = −0.473) and higher age (*β* = −0.365). Less education (*β* = 0.403) and a higher UPDRS-III score at baseline (*β* = −0.328) predicted a decrease of motor symptoms (UPDRS-III).

Lower cognitive baseline scores were also the most frequently observed predictors for training gains in CG (Table 2).

## 4. Discussion

The main aim of this multicenter RCT was to investigate the effects of a 6-week multidomain group CT compared to physical training as an active CG in PD-MCI patients. Main results are that (i) CT is feasible and safe for PD-MCI patients, (ii) CT enhanced executive functions (especially verbal fluency) as the primary outcome (but not memory) and self-reported physical activity, while (iii) the CG intervention improved working memory, and (iv) CT effects can be predicted by the respective cognitive baseline scores and partly also by education, clinical variables, and the ApoE state.

Our finding that CT improves executive functions (with a medium effect size, statistical trend) and significantly verbal fluency (with a medium effect size) is in line with meta-analyses on CT in PD patients demonstrating significant (medium) effect sizes on executive functions [9, 10]. This effect is of high clinical relevance because executive functions are the most vulnerable cognitive function in PD-MCI [2, 35], and CT appears to be an effective strategy to strengthen this function. Moreover, verbal fluency deficits are frequent in PD patients [36] and of known relevance for the patients' QoL [37]. However, the fact that a statistical trend for an interaction effect for working memory (which is also frequently regarded as a subdomain of executive functions [38]) in favor of CG (which is mainly based on the data of the digit span backward task) was found, it challenges the CT effects' specificity, an issue which has been discussed in the literature when CT is contrasted to active CG [39]. Our CG included low-intensity physical training. Although the effects of flexibility, stretching, and relaxation on cognition are not fully clear due to very limited studies—on the basis of evidence that especially aerobic, resistance, or multimodal training including aerobic exercises lead to cognitive effects [40]—our CG training had been designed to affect cognition to a minor extent. However, physical activity may enhance working memory [41], and a recent meta-analysis even demonstrated that for older adults with and without cognitive impairment, the type of exercise (aerobic, anaerobic, multicomponent, and psychomotor) was no predictor for cognitive benefits [42]. Our finding that low-intensity training was superior regarding working memory is in line with these data. However, it should be noted that the mean scores of the two groups before and after the intervention indicate that this effect is mainly driven by a relatively worse performance in the CT group at the posttest. Furthermore, a possible reason why our CT showed effects in the overall domain of executive functions (though only with a statistical trend), assessed with tests for “classical” subfunctions, but not in working memory is that the former functions were trained in higher intensity in our CT program. Future studies with different CTs and active CG will have to elucidate this differentiated picture. Notably, the finding that both the CT and CG programs yielded cognitive effects points to the potential of nonpharmacological interventions in PD per se, so that these approaches should be investigated further and receive more consideration in clinical practice. Due to the PD patients' symptom profile, further research on combined cognitive-motor trainings may have high potential for efficient treatment [43]. Even more broadly, a comparison of effects on various outcomes including cognition and motor functions of complementary interventions which challenge cognitive functions, such as theater therapy [44, 45], Tai Chi [46], tango [47], Irish dance [48], or northern walking [49], e.g., in a network meta-analysis, could improve clinical decision-making. Unfortunately, in the field of primarily physical interventions, few studies so far included cognitive outcomes [50].

Memory was not found to be enhanced by our CT. This result was unexpected, as (i) other PD studies found effects of multidomain CT on memory [19, 31, 51], and (ii) memory was explicitly trained in our CT. Furthermore, (iii) memory training in various populations has been found to yield memory benefits [52]. One possible explanation is that the intensity of our memory training was not strong enough to induce measurable improvement. Notably, an earlier study [31] found significant effects of NEUROvitalis on verbal learning in nondemented PD patients, but in that study, the original version of the program was used, while in the current study, a variant tailored to the typical PD cognitive profile was used in which two “memory sessions” were replaced by sessions focusing on executive functions and visuocognition. Future studies will have to explore which mixture of tasks is best suited for PD patients.

No significant effects of CT on noncognitive outcomes were observed. This result is in line with a meta-analysis [9], which also failed to demonstrate effects of CT on depression, QoL, and ADL. Future studies should include further patient-reported outcome measures (PROMs) and patient-reported experience measures (PREMs) [53], which are increasingly regarded as relevant to evaluate intervention effects and might be sensitive to change.

Our predictor analysis of CT effects points to the fact that the strongest cognitive benefits can be reached in vulnerable patients in terms of the level of cognitive functioning, education, disease progression, and ApoE state. The most consistent result is that low cognitive baseline scores in the respective cognitive domain are predictive for larger improvement. Notably, this was partly also shown for CG, questioning the specificity of this result. The statistical “regression to the mean” effect and retest effects might also partly contribute to these findings. However, as both groups performed comparable at baseline, patients were randomly assigned to CT or CG, and each cognitive domain was assessed with several neuropsychological tests; the possibility for regression to the mean effects (for both training effect analyses by MANOVAs and ANOVAs and prediction analyses by linear regressions) was already reduced at the design stage [54]. Although only future studies with passive CGs will be able to rule out retest effects, this finding corroborates other studies in healthy elderly [13, 15, 18, 55, 56] and PD [19, 20] showing that those individuals with lower cognitive baseline scores profit more from CT, although opposite results have been reported [16, 21]. Notably, our finding that higher LEDD intake at baseline (and lower UPDRS-III scores which show a tendency to be related to higher LEDD intake in our sample according to a post hoc analysis; Pearson's correlation *r* = −0.23, *p*=0.23) predicts a better outcome in executive functions (fluency tasks) suggest that those patients with a more pronounced levodopa effect in the motor domain are those who show more cognitive gains. Finally, carrying the ApoE4 allele was a predictor for more benefits in executive function and language. This pattern was unexpected because the ApoE4 allele has been reported to be a negative predictor of cognitive gains in healthy elderly [18] and (non-PD) MCI patients [57]. ApoE4 is a risk factor for Alzheimer's disease and also for cognitive decline in PD [58] and has been related to reduced cognitive functions and cognitive plasticity [59]. Therefore, although our results are consistent in that the more vulnerable patients are those who profit more from CT, the literature on ApoE4 effects on CT gains is inconsistent and will have to be investigated further.

Clinically, although a recent study conducting working memory training in PD patients without cognitive impairment found the opposite (i.e., “fitter” patients with higher education, better cognitive function, and shorter disease duration profit more) [21], our results may indicate a more urgent requirement for intervention in PD-MCI patients who are at more advanced stages in terms of cognitive decline and disease stage [60].

Several limitations have to be considered when interpreting our results. First, even though this was a multicenter trial, we were unable to recruit the sample size of *n* = 68 to achieve 80% power. Recruitment difficulties included the relatively long and intensive intervention and mobility constraints. However, with our *n* = 61, a post hoc power analysis still revealed 76.8% power to detect medium-sized effects for the time × group interaction effects. Second, a passive CG was not included. One could argue that the active CG is the strength of our study because the benefits shown cannot be assigned to unspecific effects (e.g., taking care of patients). However, because “no treatment with CT” reflects the clinical routine, the comparison to no treatment would have higher clinical relevance [60]. Future studies with three-arms (CT, active, and passive CGs) [31] would be reasonable. Notably, blinding of the outcome measures at the posttest was not complete as some patients reported about their interventions even though they were instructed not to do so. However, this is a more general problem of nonpharmacological studies. No information on cognitively stimulating activities was recorded, so that corresponding effects cannot be ruled out. In further, trials, e.g., diaries to control for this aspect should be included. The multidomain CT used in this study does not allow an analysis of specific factors that determine benefits. However, while more targeted interventions (e.g., working memory training) may be more effective to enhance a specific function, multidomain CT has been recommended (e.g., for individuals with (non-PD) MCI [61]) because it covers more than one critical function in the target group, and the PD-MCI cognitive profile is diverse [62]. It can be questioned whether our study that used the specific program NEUROvitalis can be generalized, and the probable answer is “no.” Importantly, CTs used in PD have been heterogeneous (individualized, computerized approaches vs. paper and pencil group interventions; different intensities) [9]. Therefore, while positive results gained from single studies (including ours) will have to be replicated in patients with different cognitive profiles (PD, PD-MCI, and PDD), it is important that the overall evidence indicates that different CTs are effective in PD patients [9, 10]. Regarding our CT, it was tailored to the PD patients' specific profile, so that it may be specifically suited for this group (although memory might have to be considered more). Also, it follows a predefined manual and is, as a published CT, available for all clinicians. These are important characteristics for a clinical implementation. Future research will have to define for which PD patients in which setting which CT is best [43].

A strength of this study is that it is one of the first multicenter RCTs on CT in PD-MCI patients diagnosed according the Level-II MDS criteria. Furthermore, our study analyses predictors of CT success. Such analyses contribute to a deeper understanding of CT mechanisms and will ultimately help to tailor interventions to individual neuropsychological profiles.

## 5. Conclusions

In conclusion, our data provide evidence that the multidomain group CT is an effective approach to treat executive function (but not memory) in PD-MCI patients, particularly in vulnerable individuals. Taking these data and the other evidence available into account [9, 10], CT should be considered as a routine treatment option for PD patients with or at risk for cognitive dysfunction. Future studies with longitudinal data are required to determine whether CT is also suitable to prevent (further) cognitive decline in PD-MCI patients and which factors determine long-term benefits. Furthermore, studies are necessary to determine which specific CT shows best effects for which PD patients. Also, health-economic analyses are necessary to evaluate the cost-effectiveness of CT in PD patients.

## Figures and Tables

**Table 1:** Training effects for cognitive outcomes for both groups at the pre- and posttest.

	Cognitive training (*n* = 33)		Physical activity (*n* = 31)		ITT − *p* value (time × group)	*η*_*p*_^2^	PP − *p* value (time × group)	*η*_*p*_^2^
	Pretest	Posttest	Pretest	Posttest				
*Global cognition*					0.322	0.287	0.166	0.374
*Memory*					0.171	0.081	0.159	0.091
CVLT total score trials 1-5^a^	−1.38 ± 1.31^1^	−0.94 ± 1.33^1^	−1.00 ± 1.19^3^	−0.91 ± 1.08^3^	0.166	0.031	0.135	0.039
CVLT long delay free recall II^a^	−1.25 ± 1.17^1^	−1.23 ± 1.37^1^	−1.18 ± 1.04^3^	−0.86 ± 1.29^3^	0.187	0.028	0.202	0.029
ROCFT delayed recall^a^	0.15 ± 0.94^1^	0.38 ± 0.91^1^	0.23 ± 1.18^2^	0.25 ± 0.88^2^	0.386	0.012	0.418	0.012
*Executive functions*					0.095	0.132	0.087	0.142
Semantic word fluency^a^	0.48 ± 0.89^1^	0.53 ± 1.13^1^	0.35 ± 1.43^2^	0.28 ± 1.42^2^	0.649	0.004	0.531	0.007
Phonemic word fluency^a^	0.27 ± 0.86^1^	0.65 ± 0.93^1^	0.34 ± 1.16^2^	0.17 ± 1.16^2^	0.018	0.092	0.020	0.094
MCST categories^a^	−0.81 ± 1.01^3^	−0.46 ± 1.01^3^	−0.92 ± 1.02^3^	−0.99 ± 0.96^3^	0.131	0.039	0.127	0.042
Key search—raw score^a^	11.42 ± 3.15^1^	11.52 ± 2.87^1^	11.13 ± 3.44^2^	10.29 ± 2.92^2^	0.320	0.017	0.250	0.024
*Attention*					0.401	0.030	0.392	0.033
d2-R errors^a^	−0.29 ± 1.40^1^	0.01 ± 1.32^1^	−0.45 ± 1.27^2^	0.06 ± 1.38^2^	0.509	0.007	0.378	0.014
d2-R concentration performance^a^	−1.57 ± 0.97^1^	−1.33 ± 0.96^1^	−1.53 ± 1.05^2^	−1.35 ± 1.05^2^	0.373	0.016	0.413	0.012
*Working memory*					0.174	0.056	0.175	0.059
Letter-number sequencing (WAIS)^a^	0.06 ± 0.77^1^	0.19 ± 0.74^1^	0.13 ± 1.02^2^	0.21 ± 1.09^2^	0.772	0.001	0.774	0.001
Digit span backward (WAIS)^a^	−0.04 ± 1.13^1^	−0.38 ± 0.86^1^	−0.42 ± 1.09^2^	−0.31 ± 0.99^2^	0.098	0.043	0.099	0.046
*Visuocognition*					0.597	0.017	0.635	0.016
ROCFT figure copy^a^	0.40 ± 1.10^1^	0.76 ± 0.95^1^	0.08 ± 1.78^2^	0.64 ± 1.20^2^	0.431	0.010	0.462	0.009
Benton judgment of line orientation^a^	−0.56 ± 1.46^1^	−0.19 ± 0.99^1^	−0.67 ± 1.35^2^	−0.13 ± 1.22^2^	0.545	0.006	0.574	0.005
*Language*					0.319	0.037	0.317	0.040
Boston naming test^a^	0.05 ± 1.18^1^	0.24 ± 1.16^1^	−0.23 ± 1.44^2^	0.19 ± 1.00^2^	0.341	0.015	0.337	0.016
Speech comprehension (ACL)—raw score^a^	17.88 ± 0.42^1^	17.82 ± 0.53^1^	17.39 ± 1.89^2^	17.81 ± 0.75^2^	0.161	0.031	0.160	0.034
*PD-MCI*								
Yes (%)	33 (100%)	21 (63.6%)	31 (100%)	20 (64.5%)				
No (%)	0 (0%)	12 (36.4%)	0 (0%)	11 (35.5%)				
Single-domain PD-MCI (%)	2 (6.1%)	1 (3.0%)	0 (0%)	0				
Multidomain PD-MCI (%)	31 (93.9%)	32 (97.0%)	31 (100%)	20 (100%)				

The references of the neuropsychological and clinical assessments are obtainable by the authors upon request; data are indicated as mean standardized *z*-scores (if available) or raw scores and standard deviations. ACL, Aphasia Check List; BDI-II, Beck Depression Inventory II; CVLT, California Verbal Learning Test; ITT, intention-to-treat analysis; MCST, Modified Wisconsin Card Sorting Test; PD-MCI, Parkinson's disease with mild cognitive impairment; PP, per-protocol analysis; ROCFT, Rey–Osterrieth Complex Figure Test; WAIS, Wechsler Adult Intelligence Scale. ^a^Higher values indicate better performance; ^1^*n* = 33; ^2^*n* = 31; ^3^*n* = 30.

**Table 2:** Exploratory analysis of training effects for noncognitive outcomes for both groups at the pre- and posttest.

	Cognitive training (*n* = 33)		Physical activity (*n* = 31)		ITT − *p* value (time x group)	*η*_*p*_^2^	PP − *p* value (time x group)	*η*_*p*_^2^
	Pretest	Posttest	Pretest	Posttest				
*Activities of daily living*								
Bayer ADL^b^	2.81 ± 2.10^2^	3.65 ± 4.34^2^	2.90 ± 2.03^4^	2.87 ± 1.67^4^	0.266	0.021	0.949	0.000
*Self-reported physical activity*								
PASE^a^	126.65 ± 72.91^1^	149.74 ± 89.20^1^	119.37 ± 62.62^3^	116.96 ± 67.16^3^	0.103	0.042	0.104	0.050
*Depression*								
BDI-II^b^	9.12 ± 5.54^1^	9.30 ± 5.82^1^	8.23 ± 4.44^3^	9.32 ± 5.38^3^	0.393	0.012	0.380	0.014
*Quality of life*								
PDQ-39^b^	36.78 ± 19.57^2^	35.72 ± 22.89^2^	35.00 ± 22.29^3^	34.39 ± 21.19^3^	0.873	0.000	0.848	0.001
*Self-experienced deficits of attention*					0.662	0.026	0.907	0.010
FEDA subscore I^a^	48.39 ± 8.70^1^	47.24 ± 13.77^1^	48.32 ± 11.79^3^	48.03 ± 12.44^3^	0.659	0.003	0.571	0.006
FEDA subscore II^a^	27.52 ± 6.63^1^	28.45 ± 7.22^1^	27.63 ± 6.43^4^	27.57 ± 6.56^4^	0.461	0.009	0.859	0.001
FEDA subscore III^a^	22.88 ± 4.10^1^	22.79 ± 4.99^1^	23.43 ± 4.15^4^	23.90 ± 3.87^4^	0.500	0.008	0.967	0.000
*Motor scores*								
UPDRS-III^b^	26.52 ± 13.88^1^	26.58 ± 12.34^1^	26.65 ± 13.41^3^	28.03 ± 14.42^3^	0.502	0.007	0.510	0.007
UPDRS-IV^b^	1.27 ± 2.30^1^	0.82 ± 1.83^1^	1.77 ± 2.67^3^	1.90 ± 2.96^3^	0.163	0.031	0.604	0.005
FOG^b^	8.24 ± 6.00^1^	9.91 ± 7.62^1^	5.97 ± 4.01^3^	7.13 ± 6.17^3^	0.712	0.002	0.965	0.000

The references of the neuropsychological and clinical assessments are obtainable from the authors upon request; data are indicated as mean standardized *z*-scores (if available) or raw scores and standard deviations. ADL, activities of daily living; FEDA, self-perceived deficits in attention; FOG, Freezing of Gait Questionnaire; ITT, intention-to-treat analysis; PASE, Physical Activity Scale for the Elderly; PP, per-protocol analysis; UPDRS, Unified Parkinson's Disease Rating Scale. ^a^Higher values indicate better performance; ^b^lower scores indicate better performance; ^1^*n* = 33; ^2^*n* = 32; ^3^*n* = 31; ^4^*n* = 30.

**Table 3:** Baseline demographic, clinical, neuropsychological, and genetic characteristics of the PD-MCI study sample.

	Cognitive training (*n* = 33)	Physical activity (*n* = 31)	*p* value
Age (years)	67.70 ± 7.19	67.52 ± 8.32	0.926
Gender			0.081
Male (%)	24 (72.7%)	16 (51.6%)	
Female (%)	9 (27.3%)	15 (48.4%)	
Years of education	13.00 (2.00–20.00)	12.00 (9.00–20.00)	0.765
Family status			0.680
Single (%)	0 (0%)	1 (3.2%)	
Married/in partnership (%)	28 (84.8%)	25 (80.6%)	
Divorced/separated (%)	2 (6.1%)	3 (9.7%)	
Widowed (%)	3 (9.1%)	2 (6.5%)	
Living situation			0.514
Single household (%)	4 (12.1%)	6 (19.4%)	
Two-person household (%)	27 (81.8%)	21 (67.7%)	
Multiperson household (%)	2 (6.1%)	3 (9.7%)	
Nursing home (%)	0 (0%)	1 (3.2%)	
Age at PD symptom onset (years)	57.84 ± 9.75	59.90 ± 8.70	0.379
Age at PD diagnosis (years)	59.79 ± 9.03	60.32 ± 8.77	0.811
PD duration (months)	86.00 (10.00–361.00)	75.00 (18.00–174.00)	0.600
Hoehn–Yahr stage			0.401
1 (%)	3 (9.1%)	6 (19.4%)	
2 (%)	19 (57.6%)	19 (61.3%)	
3 (%)	10 (30.3%)	6 (19.4%)	
4 (%)	1 (3.0%)	0 (0%)	
5 (%)	0 (0%)	0 (0%)	
UPDRS-III	24.00 (6.00–63.00)	25.00 (4.00–56.00)	0.925
UPDRS-IV	0.00 (0.00–9.00)	0.00 (0.00–7.00)	0.777
LEDD	755.50 (260.00–2050.00)	715.00 (100.00–1632.50)	0.224
Antidementiva medication intake	7 (21.2%)	7 (22.6%)	0.895
ApoE4 carriers	6 (18.2%)^a^	3 (9.7%)^b^	0.384
MoCA (max. 30 points)	25.00 (16.00–28.00)	25.00 (15.00–30.00)	0.419
SCI—number of impaired cognitive domains (max. 6 points)	3.00 (0.00–6.00)	3.00 (0.00–6.00)	0.888
BDI-II (max. 63 points)	9.00 (0.00–19.00)	8.00 (2.00–17.00)	0.499
GSE (max. 40 points)	30.13 ± 5.69	29.13 ± 5.43	0.481
PD-MCI subtype			0.164
Single-domain PD-MCI (%)	2 (6.1%)	0 (0%)	
Multidomain PD-MCI (%)	31 (93.9%)	31 (100%)	
Physiotherapy at baseline	22 (66.7%)	23 (74.2%)	0.510
Cognitive training previously	2 (6.1%)	4 (12.9%)	0.348
Self-reported activity level^c^	2.00 (1.00–3.00)	2.00 (0.00–3.00)	0.125

The references of the neuropsychological and clinical assessments are obtainable from the authors upon request. Values are presented as the mean ± standard deviation or median and range or frequency with percentages. For baseline comparison between groups, *p* values of Mann–Whitney *U* tests, independent sample *t*-tests, or *χ*^2^-tests are reported as appropriate. PD, Parkinson's disease; UPDRS, Unified Parkinson's Disease Rating Scale; LEDD, levodopa equivalent daily dose; APOE, apolipoprotein E; MoCA, Montreal Cognitive Assessment; SCI, subjective cognitive impairment questionnaire; BDI-II, Beck Depression Inventory II; GSE, General Self-Efficacy Questionnaire; MCI, mild cognitive impairment; ^a^*n* = 32; ^b^*n* = 28; ^c^self-reported activity level: 0, “not at all active;” 1, “little active;” 2, “moderate active;” 3, “very active.”

**Table 4:** Regression analyses: predictors for intervention success.

Model	Analysis	*F*-test	*R* ^2^ (adjusted *R*^2^)	Standardized coefficients (*β*) of significant predictors					
				Baseline level	Age at baseline	Education in years	UPDRS-3 at baseline	LEDD at baseline	ApoE4 carrier
*Cognitive training*									
*Memory*									
ROCFT delayed recall^a^	ITT	*F* (6, 25) = 2.688; *p*=0.037	0.392 (0.246)	−0.506^*∗∗*^^↓^	—	—	—	—	—
	PP	*F* (6, 22) = 3.618; *p*=0.012	0.497 (0.359)	−0.583^*∗∗∗*^^↓^	—	—	—	−0.373^†↓^	−0.335^†↓^
*Executive functions*									
Executive functions mean score^a^	ITT	*F* (6, 22) = 2.673; *p*=0.042	0.422 (0.264)	−0.446^*∗∗*^^↓^	—	—	—	—	—
	PP	n.s.	—	—	—	—	—	—	—
Semantic word fluency^a^	ITT	*F* (6, 25) = 2.643; *p*=0.040	0.388 (0.241)	−0.398^*∗*^^↓^	—	—	—	0.493^*∗*^^↑^	—
	PP	*F* (6, 23) = 2.865; *p*=0.031	0.428 (0.278)	−0.478^*∗*^^↓^	—	—	—	0.546^*∗*^^↑^	—
Phonemic word fluency^a^	ITT	*F* (6, 25) = 3.595; *p*=0.010	0.463 (0.334)	−0.587^*∗∗∗*^^↓^	0.405^†↑^	—	−0.507^*∗*^^↓^	0.423^*∗*^^↑^	0.473^*∗*^^↑^
	PP	*F* (6, 22) = 3.660; *p*=0.011	0.500 (0.363)	−0.592^*∗∗*^^↓^	—	−0.340^†↓^	−0.440^*∗*^^↓^	0.472^*∗*^^↑^	0.500^*∗*^^↑^
MCST categories^a^	ITT	*F* (6, 22) = 2.797; *p*=0.035	0.433 (0.278)	−0.562^*∗∗*^^↓^	−0.374^†↓^	—	—	—	—
	PP	*F* (6, 20) = 2.837; *p*=0.036	0.460 (0.298)	−0.580^*∗∗*^^↓^	—	—	—	—	—
Key search^a^	ITT	*F* (6, 25) = 2.778; *p*=0.033	0.400 (0.256)	−0.660^*∗∗∗*^^↓^	—	—	—	—	—
	PP	*F* (6, 23) = 2.651; *p*=0.042	0.409 (0.255)	−0.660^*∗∗*^^↓^	—	—	—	—	—
*Working memory*									
Working memory mean score^a^	ITT	*F* (6, 25) = 2.705; *p*=0.037	0.394 (0.248)	−0.622^*∗∗∗*^^↓^	—	—	—	—	—
	PP	*F* (6, 23) = 2.670; *p*=0.041	0.411 (0.257)	−0.633^*∗∗∗*^^↓^	—	—	—	—	—
Digit span backward (WAIS)^a^	ITT	*F* (6, 25) = 5.311; *p*=0.001	0.560 (0.455)	−0.729^*∗∗∗*^^↓^	—	—	—	—	—
	PP	*F* (6, 23) = 5.700; *p*=0.001	0.598 (0.493)	−0.744^*∗∗∗*^^↓^	—	—	—	—	—
*Visuocognition*									
Visuocognition mean score^a^	ITT	*F* (6, 25) = 5.052; *p*=0.002	0.548 (0.440)	−0.548^*∗∗*^^↓^	0.334^†↑^	—	—	—	—
	PP	*F* (6, 23) = 4.770; *p*=0.003	0.554 (0.438)	−0.552^*∗∗*^^↓^	—	—	—	—	—
ROCFT figure copy^a^	ITT	*F* (6, 25) = 3.460; *p*=0.013	0.454 (0.323)	−0.515^*∗∗*^^↓^	—	—	—	—	—
	PP	*F* (6, 23) = 3.296; *p*=0.017	0.462 (0.322)	−0.537^*∗∗*^^↓^	—	—	—	—	—
Benton judgment of line orientation^a^	ITT	*F* (6, 25) = 5.049; *p*=0.002	0.548 (0.439)	−0.722^*∗∗∗*^^↓^					
	PP	*F* (6, 23) = 5.009; *p*=0.002	0.567 (0.453)	−0.726^*∗∗∗*^^↓^	—	—	—	—	—
*Language*									
Language mean score^a^	ITT	*F* (6, 25) = 3.012; *p*=0.023	0.420 (0.280)	−0.565^*∗∗*^^↓^	—	—	−0.441^*∗*^^↓^	—	0.463^*∗*^^↑^
	PP	*F* (6, 23) = 2.786; *p*=0.035	0.421 (0.270)	−0.555^*∗∗*^^↓^	—	—	−0.392^*∗*^^↓^	—	0.407^†↑^
Speech comprehension (ACL)^a^	ITT	*F* (6, 25) = 3.842; *p*=0.007	0.480 (0.355)	−0.642^*∗∗∗*^^↓^	—	—	—	—	—
	PP	*F* (6, 23) = 3.672; *p*=0.011	0.489 (0.356)	−0.655^*∗∗∗*^^↓^	—	—	—	—	—
*Noncognitive outcomes*									
BDI-II^b^	ITT	*F* (6, 25) = 2.858; *p*=0.029	0.407 (0.264)	−0.473^*∗∗*^^↑^	−0.365^†↑^	—	—	—	—
	PP	*F* (6, 23) = 2.724; *p*=0.038	0.415 (0.263)	−0.489^*∗*^^↑^	—	—	—	—	—
UPDRS-III^b^	ITT	*F* (5, 26) = 3.281; *p*=0.020	0.387 (0.269)	−0.328^†↑^	—	0.403^*∗*^^↓^	—	—	—
	PP	*F* (5, 24) = 4.583; *p*=0.004	0.488 (0.382)	−0.372^*∗*^^↑^	—	0.467^*∗*^^↓^	—	−0.376^†↑^	—

*Control group*									
*Memory*									
CVLT total score trials 1–5^a^	ITT	*F* (6, 20) = 2.978; *p*=0.030	0.472 (0.313)	−0.702^*∗∗*^^↓^	−0.456^†↓^	—	—	—	—
	PP	*F* (6, 19) = 3.051; *p*=0.029	0.491 (0.330)	−0.690^*∗∗*^^↓^	−0.429^†↓^	—	—	—	—
CVLT long delay free recall II^a^	ITT	*F* (6, 20) = 3.219; *p*=0.022	0.491 (0.339)	−0.362^*∗*^^↓^	−0.776^*∗∗*^^↓^	−0.637^*∗∗*^^↓^	—	—	—
	PP	*F* (6, 19) = 3.042; *p*=0.029	0.490 (0.329)	−0.346^†↓^	−0.777^*∗∗*^^↓^	−0.599^*∗∗*^^↓^	—	—	—
ROCFT delayed recall^a^	ITT	*F* (6, 21) = 4.872; *p*=0.003	0.582 (0.462)	−0.527^*∗∗*^^↓^	0.462^*∗*^^↑^	—	—	—	—
	PP	*F* 6, 20) = 4.640; *p*=0.004	0.582 (0.456)	−0.523^*∗∗*^^↓^	0.459^*∗*^^↑^	—	—	—	—
*Executive functions*									
Key search^a^	ITT	*F* (6, 21) = 4.075; *p*=0.007	0.538 (0.406)	−0.780^*∗∗∗*^^↓^	—	—	—	—	—
	PP	*F* (6, 20) = 3.942; *p*=0.009	0.542 (0.404)	−0.785^*∗∗∗*^^↓^	—	—	—	—	—
*Visuocognition*									
Visuocognition mean score^a^	ITT	*F* (6, 21) = 2.972; *p*=0.029	0.459 (0.305)	−0.654^*∗∗∗*^^↓^	—	—	—	—	—
	PP	*F* (6, 20) = 3.285; *p*=0.020	0.496 (0.345)	−0.667^*∗∗∗*^^↓^	—	—	—	—	—
ROCFT figure copy^a^	ITT	*F* (6, 21) = 5.361; *p*=0.002	0.605 (0.492)	−0.796^*∗∗∗*^^↓^	—	—	—	—	—
	PP	*F* (6, 20) = 5.638; *p*=0.001	0.628 (0.512)	−0.802^*∗∗∗*^^↓^	—	—	—	—	—
*Language*									
Language mean score^a^	ITT	*F* (6, 21) = 9.890; *p* < 0.001	0.739 (0.664)	−0.847^*∗∗∗*^^↓^	—	—	—	—	—
	PP	*F* (6, 19) = 9.417; *p* < 0.001	0.748 (0.669)	−0.856^*∗∗∗*^^↓^	—	—	—	—	—
Boston naming test^a^	ITT	*F* (6, 21) = 6.582; *p*=0.001	0.653 (0.554)	−0.678^*∗∗∗*^^↓^	—	—	—	—	—
	PP	*F* (6, 20) = 6.563; *p*=0.001	0.663 (0.562)	−0.619^*∗∗∗*^^↓^	—	—	—	—	—
Speech comprehension (ACL)^a^	ITT	*F* (6, 21) = 25.963; *p* < 0.001	0.881 (0.847)	−0.900^*∗∗∗*^^↓^	—	—	—	−0.196^*∗*^^↓^	—
	PP	*F* (6, 19) = 23.387; *p* < 0.001	0.881 (0.843)	−0.899^*∗∗∗*^^↓^	—	—	—	−0.187^†↓^	—

Dependent variables are defined as Δposttest − pretest; only those regression models that reached statistical significance at *p* < 0.05 are presented; for each significant regression model, standardized regression coefficients are reported for predictors that have reached statistical significance only; ^†^*p* ≤ 0.10; ^*∗*^*p* ≤ 0.05; ^*∗∗*^*p* ≤ 0.01; ^*∗∗∗*^*p* ≤ 0.001. ACL, Aphasia Check List; ApoE, apolipoprotein E; BDI, Beck Depression Inventory; CVLT, California Verbal Learning Test; ITT, intention-to-treat analysis; LEDD, levodopa equivalent daily dose; MCST, Modified Wisconsin Card Sorting Test; PP, per-protocol analysis; ROCFT, Rey–Osterrieth Complex Figure Test; UPDRS, Unified Parkinson's Disease Rating Scale; WAIS, Wechsler Adult Intelligence Scale. ^a^Higher change scores indicate higher training gains; ^b^lower change scores indicate higher training gains. ^↓^Lower predictor level predicts higher training gains. ^↑^Higher predictor level predicts higher training gains.

## Data Availability

The data used to support the findings of this study are available from the corresponding author upon request.

## Supplementary Materials

Supplementary Materials Supplementary Table 1: characteristics of the interventions. Supplementary Table 2: feasibility analysis: participation score, patients' satisfaction, and transfer to daily life. Table 3: CONSORT 2010 checklist of information to include when reporting a randomized trial.
