# Supplementary material for: Enhancement of Executive Functions but Not Memory by Multidomain Group Cognitive Training in Patients with Parkinson's Disease and Mild Cognitive Impairment: A Multicenter Randomized Controlled Trial
Source: Parkinsons Dis. 2020 Nov 30;2020:4068706. doi: 10.1155/2020/4068706 (PMC7721510; doi:10.1155/2020/4068706)
Supplement: Supplementary Materials — Supplementary Table 1: characteristics of the interventions. Supplementary Table 2: feasibility analysis: participation score, patients' satisfaction, and transfer to daily life. Table 3: CONSORT 2010 checklist of information to include when reporting a randomized trial. [file 4068706.f1.zip › 4068706.f1/Supplementary Table 1_Interventions.docx]

**Supplementary Table 1. Characteristics of the interventions**

|  | **Cognitive training NEUROvitalis** | **Physical activity (control condition)** |
| --- | --- | --- |
| **Basic characteristics** | - Developed by neuropsychologists; - Published material with a detailed manual explaining the structure and content of each NEUROvitalis session (Baller et al., 2009) | - Developed specifically as a control condition for this study by a sports scientist (author CS) - Comprises a detailed manual explaining structure and content of each session |
| **Aims/target groups** | - Cognitive multidomain training targeting specific cognitive functions - Target groups: healthy elderly, individuals at risk for cognitive dysfunctions, MCI patients - Development of a PD expansion module to target vulnerable cognitive functions in a balanced way (more executive functions and visuocognition compared to the original version) - Two level of severity to adapt for patients’ cognitive profile | - Physical training beneficial for PD symptoms but expecting to have minor cognitive effects (no endurance and strength training) - Training intensity had to be less than 50% of the heart rate reserve |
| **Frequency and intensity** | 12 sessions two times weekly for 90 minutes | |
| **Group size** | Three to five patients | |
| **Intervention facilitators** | - Psychologists, gerontologists trained for conducting the program | - Physiotherapists and move gerontologists with experience in working with PD patients and trained for the program |
| **Training elements (part of every training session)** | - Psychoeducation (20 min) - Group tasks and activity games (60 min) - Individual exercises (10 min) - Homework | - Warm up (15 min) - Exercises on stretching, flexibility, loosening up, and relaxation (55 min) - Psychoeducation (20 min) - Homework |
| **Main domains trained** | - Executive functions - Memory - Attention - Visuocognition | - Stretching - Flexibility - Loosening up - Relaxation |
| **Topics of psychoeducational part** | 1. Cognitive impairment and treatment options in PD 2. Attention 3. Memory 4. Working memory 5. Executive functions I: planning and problem solving 6. Memory strategies I: repeating and clustering 7. Memory strategies II: information storage with the help of different sensory channels and story method 8. Memory for names and faces 9. Memorizing dates and to do’s 10. Visuocognition 11. Executive functions II: social cognitive and emotional executive functions 12. Protective and risk factors for preserving the cognitive performance in old age   *Handouts with detailed information on the topics and cognitive exercises were given to the participants* | 1. Background information on PD 2. PD therapy options 3. Deep brain stimulation 4. Exercise therapy 5. How to get PD-specific information 6. Freezing 7. Nutrition I 8. Nutrition II 9. Swallowing dysfunctions in PD 10. Occupational therapy 11. Therapeutic appliances in PD 12. Fall risk in PD   *Handouts with detailed information on the topics were given to the participants* |

*Abbreviations:* PD = Parkinson’s disease; MCI = Mild cognitive impairment
