# Supplementary material for: Enhancement of Executive Functions but Not Memory by Multidomain Group Cognitive Training in Patients with Parkinson's Disease and Mild Cognitive Impairment: A Multicenter Randomized Controlled Trial
Source: Parkinsons Dis. 2020 Nov 30;2020:4068706. doi: 10.1155/2020/4068706 (PMC7721510; doi:10.1155/2020/4068706)
Supplement: Supplementary Materials — Supplementary Table 1: characteristics of the interventions. Supplementary Table 2: feasibility analysis: participation score, patients' satisfaction, and transfer to daily life. Table 3: CONSORT 2010 checklist of information to include when reporting a randomized trial. [file 4068706.f1.zip › 4068706.f1/Supplementary Table 2_Feasbility analysis.docx]

**Supplementary Table 2. Feasibility analysis: Participation score, patients’ satisfaction and transfer to daily life**

|  | **Cognitive training**  **(*n* = 31)** | **Physical activity**  **(*n* = 30)** | ***p* value** |
| --- | --- | --- | --- |
| Participation score | 11.00 (8.00-12.00) | 11.00 (6.00-12.00) | .126 |
| How motivated are you to participate in the training session?^1^ | 4.00 (3.00-5.00) | 4.00 (3.00-5.00) | .734 |
| How did you like the training session?^2^ | 4.00 (3.00-5.00) | 5.00 (4.00-5.00) | **.001** |
| How often did you continue to train at home? (max. 11 times) | 9.00 (4.00-11.00) | 6.00 (0.00-11.00) | **.045** |
| Grade for the overall training^3^ | 2.00 (1.00-3.00) | 2.00 (1.00-3.00) | .903 |
| Would you recommend the training? | Yes: 100% | Yes: 100% | 1.000 |

Values are presented as median and range or percentages. For comparison between groups, *p*-values of Mann-Whitney-U tests or χ^2^-tests are reported as appropriate.

^1^ 6-point Likert scale from 0 = “not motivated” to 6 = “very motivated”.

^2^ 6-point Likert scale from 0 = “not good at all” to 6 = “very good”.

^3^ Grades in accordance with the German grading system ranging from 1 = „very good“ to 6 = “insufficient”.
